# Posterior shoulder dislocation with reverse Hill-Sachs lesion. A technical note and report of two cases

**DOI:** 10.1051/sicotj/2021022

**Published:** 2021-03-26

**Authors:** Konstantinos Kazamias, Vasiliki Bisbinas, George Markopoulos, Stavros Pellios, Ilias Bisbinas

**Affiliations:** 1 Lieutenant and Resident in Orthopaedics and Trauma Surgery, Department of 1st Orthopaedic and Trauma, 424 Army General Training Hospital 56429 Thessaloniki Greece; 2 S.H.O. in Orthopaedics and Trauma, Department of Orthopaedic and Trauma, Conquest Hospital Hastings TN37 7RD East Sussex UK; 3 Major and Consultant in Orthopaedics and Trauma Surgery, Department of 1st Orthopaedic and Trauma, 424 Army General Training Hospital 56429 Thessaloniki Greece; 4 Col and Consultant in Orthopaedics and Trauma Surgery, Chief of the Department of 1st Orthopaedic and Trauma, 424 Army General Training Hospital 56429 Thessaloniki Greece

**Keywords:** Posterior shoulder dislocation, Reverse Hill-Sachs, Surgery

## Abstract

Posterior shoulder dislocation (PSD) with a reverse Hill-Sachs lesion is a rare injury with challenging management. This article is a technical note, describing the combination of both, modified McLaughlin procedure with posterior Bankart repair, for the surgical treatment of traumatic PSD associated with a substantial reverse Hill-Sachs lesion. Two patients with mid-term follow-up are presented. Approaching and repairing both sides of the joint, balance and congruency are restored, the humeral head is centralized in the glenoid and the patient starts early mobilization and rehabilitation safely.

## Introduction

A posterior shoulder dislocation (PSD) associated with reverse Hill-Sachs lesion is a rare injury, often missed or misdiagnosed, and CT and MRI scans are needed to detect the associated bone and soft tissue lesions [1–3]. Treatment should be individualized taking into account the patient’s features as well as bone and soft tissue lesions in both sides of the shoulder joint, humeral head, and glenoid [4].

This article describes the application of both together, modified McLaughlin procedure and posterior Bankart repair, and presents two patients with mid-term clinical outcome. Although both procedures are not always combined in the literature, to our knowledge, our cases are the first ones in the literature documenting that modified McLaughlin procedure potentially is not enough, confirming that the clinical outcome is more reliable after the combination of both procedures [4–6].

## Material and methods

Two patients, a 30 and 52-year-old, are presented with a PSD, both after trauma – a simple fall on the floor. The first patient with an acute injury of his right dominant shoulder and the second one, with a four months history of “locked” reverse Hill-Sachs lesion of his left non-dominant shoulder, was treated non-operatively elsewhere without him to be satisfied regarding pain and ROM.

**Figure 1:**
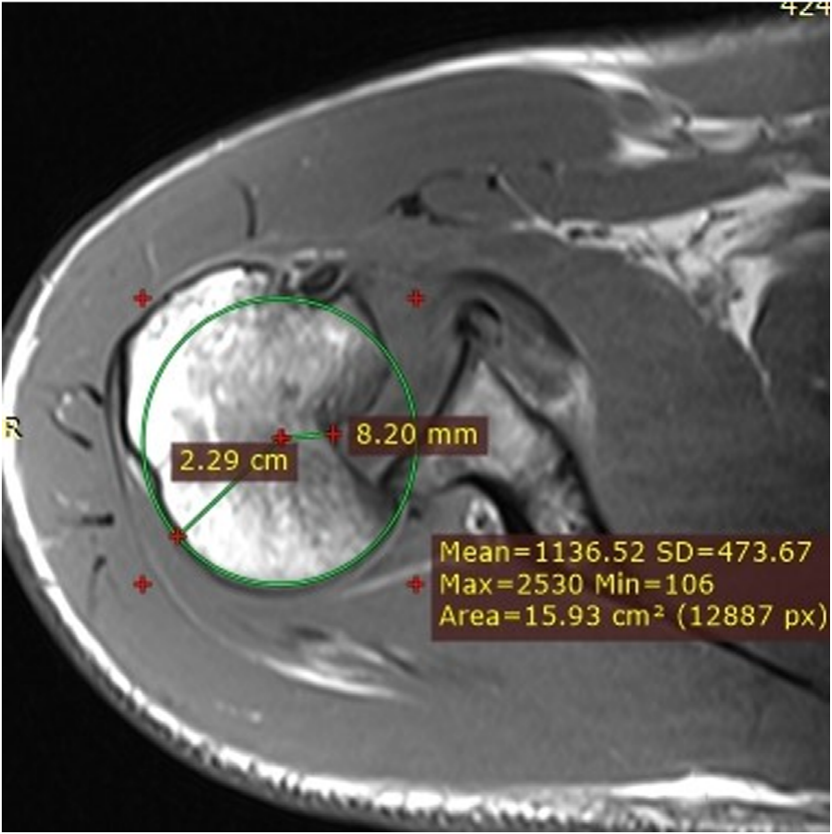
1st patient: MRI scan showing PSD, reverse Hill-Sachs lesion, impaction involving approximately 40% of the articular surface of the humeral head, and an intact posterior glenoid rim.

**Figure 2:**
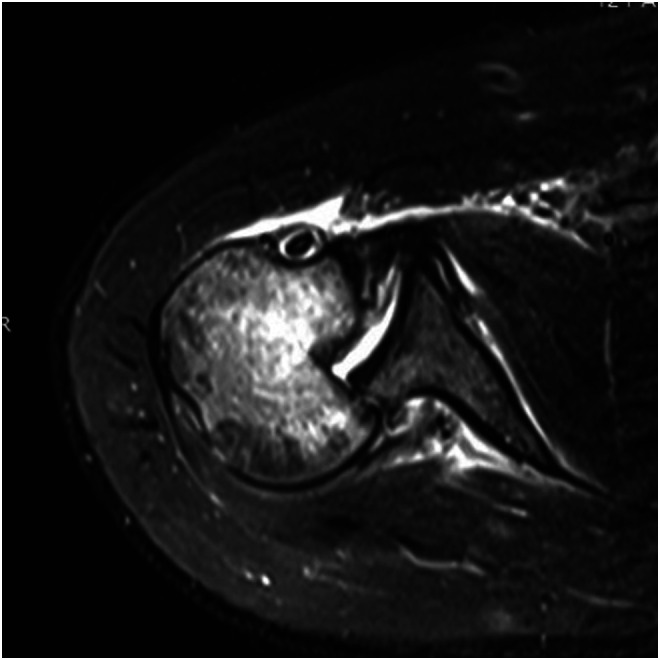
1st patient: MRI scan showing PSD, reverse Hill-Sachs, reverse Bankart lesion, intact posterior glenoid rim, intact lesser tubercle, and subscapularis tendon.

**Figure 3:**
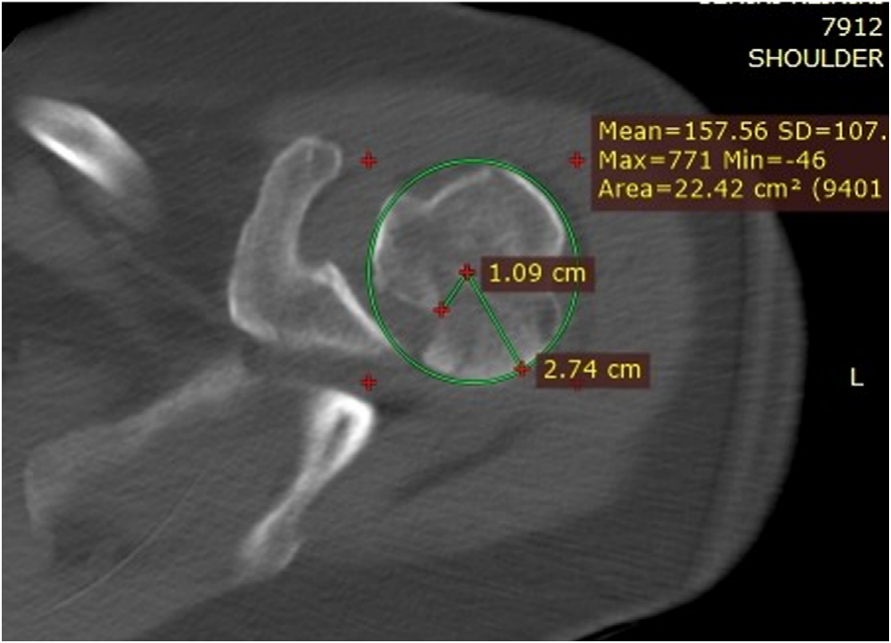
2nd patient: CT scan confirming PSD with associated reverse Hill-Sachs lesion involving approximately 40% of the articular surface of the humeral head.

**Figure 4:**
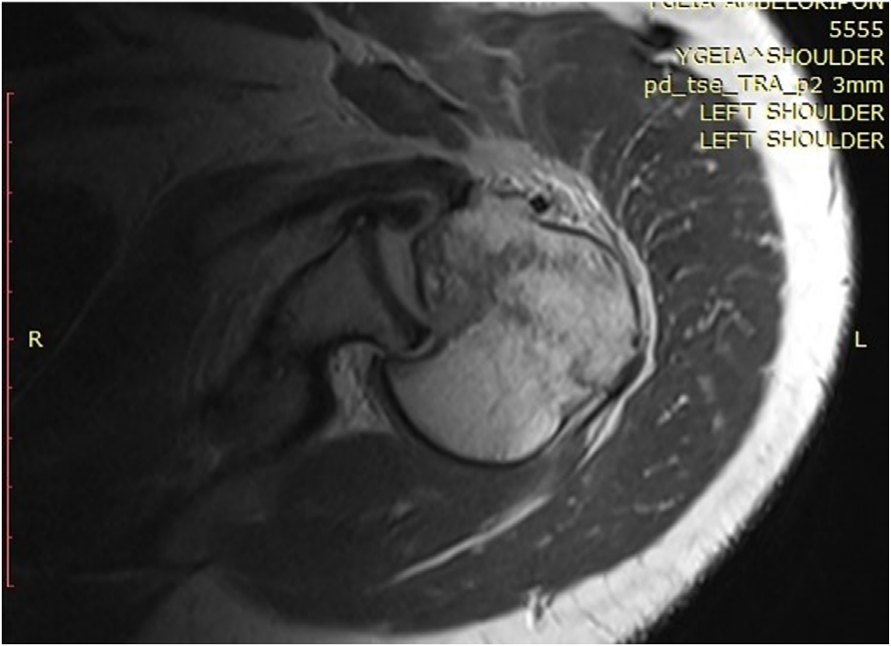
2nd patient: MRI scan showing PSD, reverse Hill-Sachs, minimally displaced humeral head fracture, reverse Bankart lesion, intact posterior glenoid rim, intact lesser tubercle, and subscapularis.

Clinical examination, plain radiographs, CT, and MRI scans in both patients, confirmed a PSD with associated reverse Hill-Sachs lesion involving approximately 40% of the articular surface of the humeral head (Figures 1 and 2). In the second patient, there was an associated healed humeral head-neck fracture malunited in approximately 20° of internal rotation (Figures 3 and 4).

In both patients, surgical repair using a modified McLaughlin procedure was decided and was applied as the main plan. Lesser tubercle osteotomy was performed and it was transferred (with the attached subscapularis) and fixed into the reverse Hill-Sachs lesion after the appropriate local disimpaction. Moreover, in the first patient, in the residual step-gap between the transferred lesser tubercle and the articular surface of the humeral head, the subscapularis tendon with the capsule was tenodesed getting the tubercle extra-articular. Immediately postoperatively, in theatre, an abduction-neutral rotation brace of the shoulder was applied.

**Figure 5:**
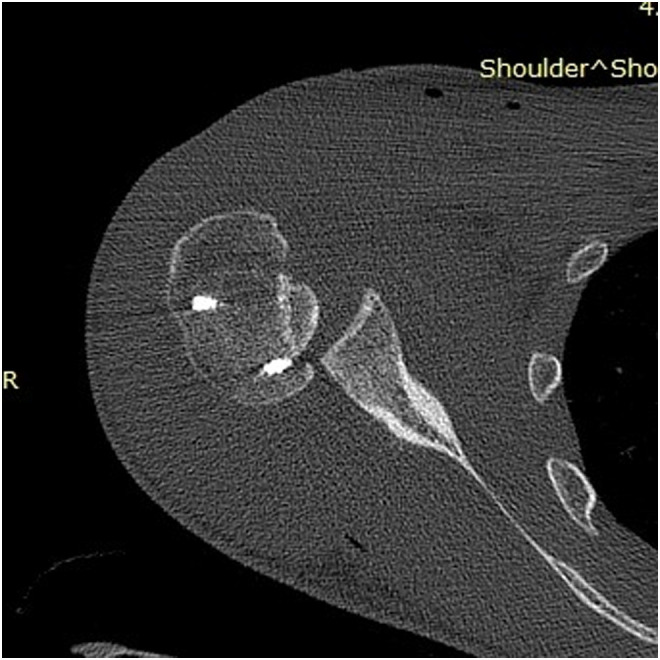
1st patient: Postoperative CT scan confirming the persistence of subluxation between reverse Hill-Sachs lesion and posterior Bankart lesion – after the modified McLaughlin procedure, in which only the humeral head’s side was addressed.

**Figure 6:**
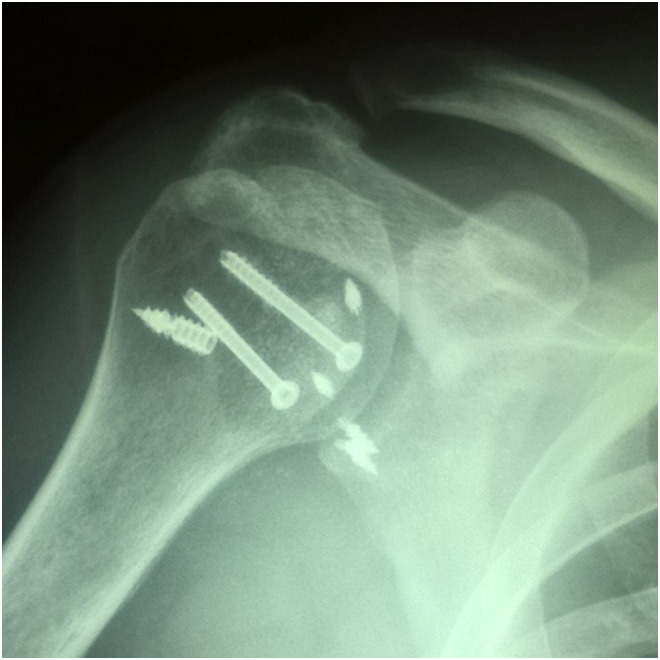
1st patient: Postoperative Right shoulder (AP) radiographs, after the second operation of shoulder arthroscopy and reverse Bankart repair with two metal anchors in posteroinferior glenoid rim.

**Figure 7:**
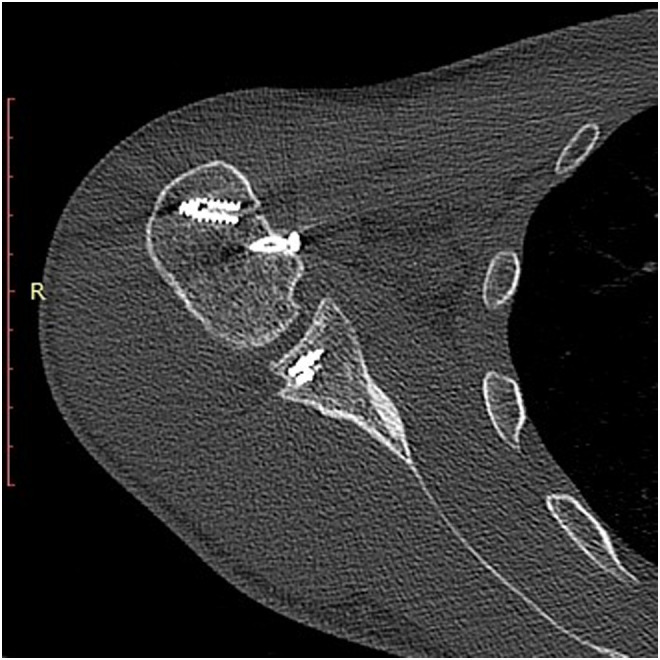
1st patient: Postoperative CT scan of Right shoulder, after the second operation of arthroscopy and reverse Bankart repair. After addressing both sides of the joint, the humeral head is centralized in the glenoid.

Unfortunately, postoperative radiographs and CT scans in the first patient showed a posterior subluxation of the humeral head engaging in the posterior glenoid rim (Figure 5). With an arthroscopy during the same admission, posterior Bankart repair was performed, using two 3 mm Ti anchors and after the second operation, radiographs (Figure 6) and CT scan (Figure 7) confirmed joint congruence and centralization of the humeral head in the glenoid.

**Figure 8:**
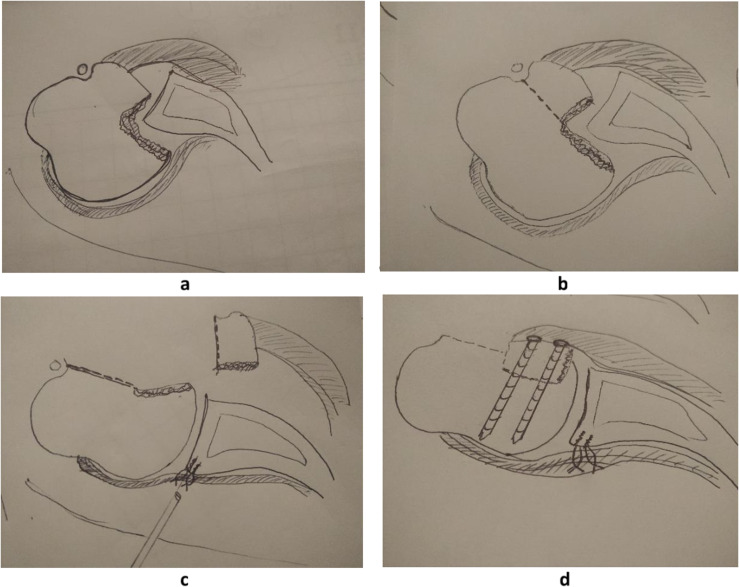
2nd patient: Technical steps applying consecutively the combined procedure. (a) Direct assessment of reverse Hill-Sachs (through a small cut in rotator cuff interval), (b) lesser tubercle osteotomy (with the attached subscapularis tendon), (c) under direct vision from the anterior joint, application of two anchors through a stab wound posteriorly, to repair the reverse Bankart lesion, and (d) lesser tubercle transfer medially in the impaction area, and fixation with screws.

In the second patient, in order to avoid residual instability, Bankart repair was applied together with modified McLaughlin procedure in the same operation. After the lesser tubercle osteotomy and before its transfer, while having an approach to the posterior glenoid, using a stab wound from posteriorly, two all-suture 2.9 mm anchors were applied to re-attach the posterior capsule (Figure 8). Combining both procedures joint congruency and stability were achieved and confirmed intraoperatively.

## Results

**Figure 9:**
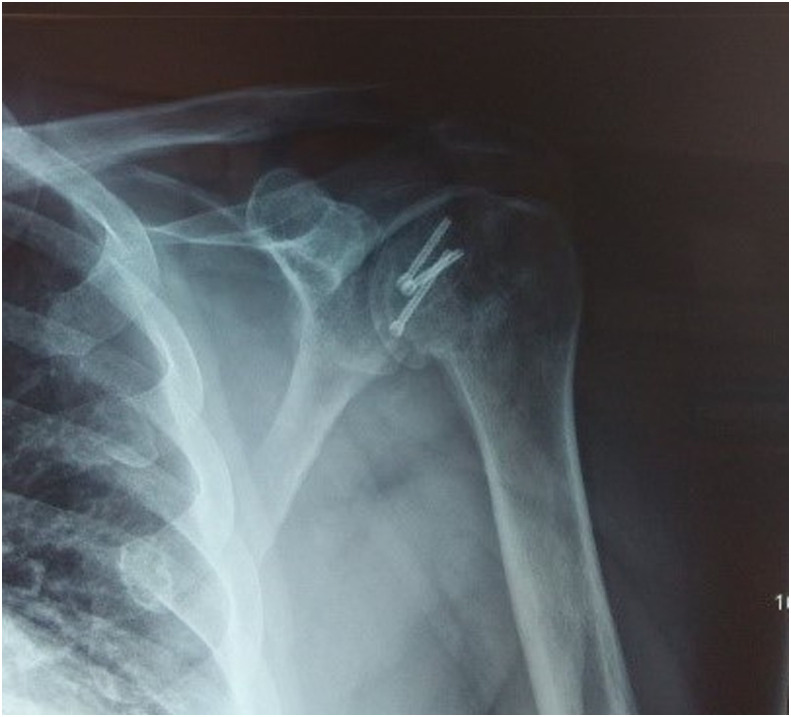
2nd patient: Postoperative (AP) radiographs of Left shoulder. Metal screws were used for lesser tubercle transfer-fixation.

**Figure 10:**
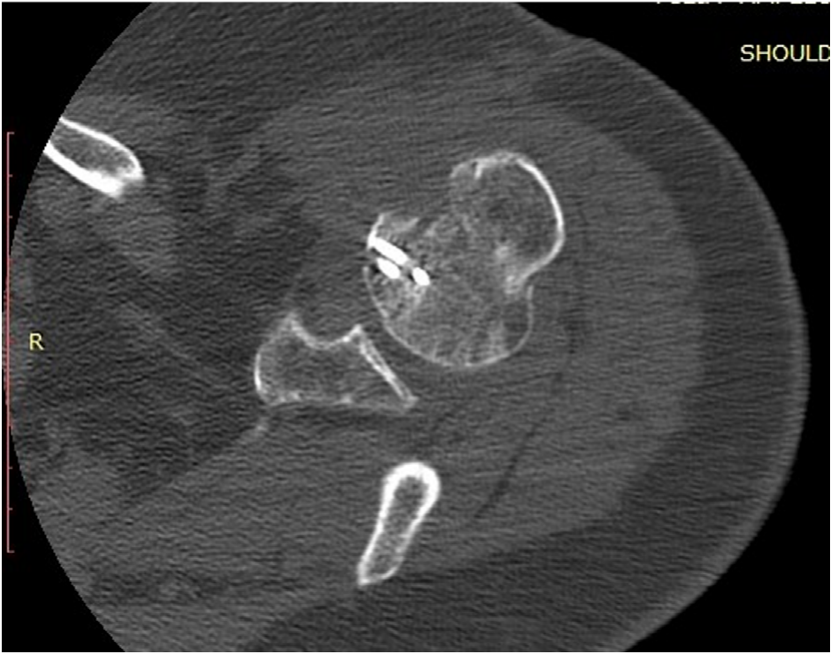
2nd patient: Postoperative CT scan of Left shoulder, after modified McLaughlin procedure and reverse Bankart repair. Humeral head is centered in the glenoid. A gap after lesser tubercle transfer is obvious-left behind. The suture anchor used for the reverse Bankart repair is not visible in CT sections.

In both patients intraoperatively, after performing only the modified McLaughlin procedure, there was a tendency for posterior instability in 10°–20° of internal rotation of the humeral head. However, it was thought that by applying an abduction-neutral rotation brace immediately postoperatively, the joint would be kept in place. In the first patient, it was proved that brace was not enough, the joint was subluxated postoperatively and a second operation was needed to stabilize it in place (Figures 5 and 7). Message received and in the second patient, both modified McLaughlin procedure and Bankart repair were applied during the same operation. Joint stability and humeral head centralization were obviously achieved intraoperatively and there was no need for the brace. Postoperative radiographs and CT scans confirmed humeral head shape improvement and joint congruency (Figures 9 and 10).

Early physiotherapy and rehabilitation began from the 4th week postoperatively and the patients improved substantially in the following three months. Both patients, at their 15-month and 9-month postoperative follow-up respectively, were happy and asymptomatic. They had a stable joint, ROM close to normal, unrestricted activities, and a Constant score of 92 and 84 respectively (Videos 1 and 2).

## Discussion

A posterior shoulder dislocation (PSD) has an incidence of 2–4% of all shoulder dislocations and often associates with other bony or soft tissue lesions including posterior labrum/rim pathology up to 97%, humeral head-neck fractures up to 46.6%, and rotator cuff lesions up to 13% [2, 3, 7]. Either because of its complex pathology in both sides of the joint or because it is often missed in the early stages, PSD still remains a “diagnostic and management trap” [1, 2, 8, 9].

The current article describes two cases of PSD with an associated Hill-Sachs lesion involving approximately 40% of the humeral head articular surface. In both cases, surgical repair applying a modified McLaughlin procedure and posterior Bankart repair was proved to be necessary to establish congruity and joint stability.

The present study has several limitations. It is a presentation of two cases only, a certain technique combining two well-established procedures was applied in both of them, and no other patient population or techniques are presented to compare with. Additionally, both cases are males and the clinical follow-up is relatively short. However, both cases were treated by the same shoulder surgeon and in the second case, applied surgery was more completed, correcting what was considered inadequate in the first one, passing across a potentially valuable message.

Management of PSD should be individualized and parameters to take into account include the age, activity level and associated comorbidities of the patient, time from injury to presentation, and the size of the reverse Hill-Sachs lesion [10]. As in our second patient, the clinical outcome after his initial non-operative management was considered unacceptable. Non-operative treatment is an option (“supervised neglect”), especially in low-demand elderly patients with concomitant comorbidities, however, in the vast majority of the patients (the young and active ones as most of them are) various operative options have been suggested [11]. A reverse Hill-Sachs lesion with impaction of more than 20–40% of the humeral head articular surface and symptoms of posterior instability or pain, usually needs to be addressed surgically [12–14]. Bone disimpaction and fracture fixation, reconstruction with muscle-tendon transfer (McLaughlin procedure or a modified technique), rotational osteotomy, humeral bone augmentation (using autograft, allograft, or a synthetic substitute), arthroscopy and posterior Bankart repair or even joint arthroplasty, are all viable options in the acute phase, depending on the various clinical and radiological parameters [11, 15–18].

It is reported that performing a modified McLaughlin procedure is enough [13, 14]. They reported good results, in terms of joint stability, simply eliminating shoulder internal rotation postoperatively, with the use of either in an abduction brace and neutral rotation or in an external rotation brace [13, 14]. However, if joint stability and posterior labrum pathology are not addressed intraoperatively, the joint stability postoperatively relies mostly on the patient’s compliance to a shoulder brace avoiding internal rotation. It is reported that arthroscopic posterior Bankart repair is a promising option [16]. However, a substantial reverse Hill-Sachs could be “off track” in internal rotation, undermining joint stability.

In both patients presented in this article, it was made obvious that if both sides of pathology (reverse Hill-Sachs and posterior Bankart) are not addressed in the same operation appropriately, surgery could potentially fail. In both cases, a posterior Bankart repair was needed further to the modified McLaughlin procedure, in order to stabilize the joint and centralize the humeral head in the glenoid. Restoring joint congruency (osteotomy and lesser tubercle transfer, subscapularis tenodesis), and establishing soft tissue balance between both sides of the joint (posterior labrum/capsule repair) are all of equal importance for early mobilization-rehabilitation and finally a good clinical outcome.

In conclusion, PSD with a substantial reverse substantial Hill-Sachs lesion involves both sides of the joint, and both should be treated. Modified Mc Laughlin procedure addresses only the reverse Hill-Sachs lesion in the anterior part of the joint.

Posterior Bankart lesion needs repair in order to avoid potential posterior subluxation – even in a brace – postoperatively. “Repairing” both sides of the joint, congruency and stability are addressed, soft tissue balance is achieved anteriorly and posteriorly, the humeral head is centralized in glenoid, early mobilization is safe leading to a rewarding clinical outcome.

## Supplementary Material

Supplementary material of this article is available at https://www.sicot-j.org/10.1051/sicotj/2021022/olm *Video 1*. 1st patient: ROM in the 12-month postoperative follow-up. *Video 2*. 2nd patient: ROM in the 6-month postoperative follow-up.

## Declarations

### Conflict of interest

The authors declare that

- they have no conflict of interest, and
- they have full control of all primary data and they agree to allow the journal to review their data if requested.

### Patients’ consent

It is indicated that consent for publication has been obtained from both patients.
